# Comparative Clinical Profile of Postural Orthostatic Tachycardia Patients With and Without Joint Hypermobility Syndrome

**Published:** 2010-04-01

**Authors:** Khalil Kanjwal, Bilal Saeed, Beverly Karabin, Yousuf Kanjwal, Blair P Grubb

**Affiliations:** Division of Cardiology, Section of Electrophysiology, The University of Toledo Medical Center

**Keywords:** autonomic dysfunction, joint hypermobility syndrome, postural orthostatic tachycardia

## Abstract

**Background:**

Autonomic dysfunction is common in patients with the joint hypermobility syndrome (JHS). However, there is a paucity of reported data on clinical features of Postural orthostatic tachycardia syndrome (POTS) in patients suffering from JHS.

**Methods:**

This retrospective study was approved by our local Institutional Review Board (IRB). Over a period of 10 years, 26 patients of POTS were identified for inclusion in this study. All these patients had features of Joint Hypermobility Syndrome (by Brighton criterion). A comparison group of 39 patients with other forms of POTS were also followed in the autonomic clinic during the same time. We present a descriptive report on the comparative clinical profile of the clinical features of Postural Orthostatic Tachycardia patients with and without Joint Hypermobility syndrome. The data is presented as a mean±SD and percentages wherever applicable.

**Results:**

Out of 65 patients, 26 patients (all females, 20 Caucasians) had POTS and JHS. The mean age at presentation of POTS was 24±13 (range 10-53 years) vs 41±12 (range 19-65 years), P=0.0001, Migraine was a common co morbidity 73 vs 29% p=0,001. In two patients POTS was precipitated by pregnancy, and in three by surgery, urinary tract infection and a viral syndrome respectively.  The common clinical features were fatigue (58%), orthostatic palpitations (54%), presyncope (58%), and syncope (62%).

**Conclusions:**

Patients with POTS and JHS appear to become symptomatic at an earlier age compared to POTS patients without JHS. In addition patients with JHS had a greater incidence of migraine and syncope than their non JHS counterparts.

##  Introduction

Joint hypermobility syndrome (JHS) is one of the most common heritable collagen disorders [1,2]. In addition to articular manifestations these patients can present with multiple extraarticular manifestations such as cutaneous scaring, ocular lid laxity, genitourinary dysfunction, peripheral nervous disorders and chronic fatigue syndrome [1-6]. Autonomic dysfunction has been reported to occur in patients with JHS and include symptoms of syncope, presyncope, palpitations, chest discomfort, fatigue, and heat intolerance, orthostatic hypotension, postural orthostatic tachycardia syndrome, and uncategorized orthostatic intolerance [8,9]. Although, postural tachycardia syndrome (POTS) has been reported to occur in patients with JHS there is paucity of data on the clinical features and outcome of POTS in patients who suffer from JHS as opposed to those with POTS from other causes. We present a comparative clinical profile of postural orthostatic tachycardia patients with and without joint hypermobility syndrome.

## Methods

The study was a retrospective descriptive analysis of the patients followed at the University of Toledo Medical Center. Our Institutional Review Board approved the study. We identified 26 patients of POTS with preexisting JHS. Patients were diagnosed as having JHS based on their clinical findings, Brighton criterion as well as Beighton score [10,11] of ≥ 4/9. These patients were diagnosed as having POTS primarily based on their history, clinical features and findings from head upright tilt table testing (HUTS). They were diagnosed as suffering from POTS if they had symptoms (> 6 months) of orthostatic intolerance associated with a heart rate increase of 30 bpm (or rate that exceeds 120 bpm) that occurs within the first 10 minutes of standing or upright tilt, not associated with other chronic debilitating conditions such as prolonged bed rest or the use of medications known to diminish vascular or autonomic tone. A group of POTS patients (n=39) without pre-existing JHS who were being followed at our center during the same time frame was selected to serve as a control group. Some of these patients were referred from various other centers to our clinic for second opinions regarding diagnosis and management. Thus the group was not a consecutive group of patients who had been diagnosed at our center only. All of these patients were primarily seen for symptoms of orthostatic intolerance. The patient charts and physician communications and letters were reviewed and the information about the demographics, clinical features, comorbid conditions, tilt table test results were collected.

The data is observational and is presented as mean ± SD and percentages. The t test for comparison of means and chi square test for comparison of percentages was used. The statistical significance was reached at P< 0.05.

## Results

Table 1 summarizes the clinical features of patients with and without JHS. The POTS patients with JHS tended to be younger (30 ±13, range 10-53) when compared to patients without JHS (40±11, range 19-65) P< 0.05.

### Precipitating Event

The most common precipitating events for POTS in patients with JHS were pregnancy (12%), surgery and urinary tract infection (4%) each. In patients without JHS the common precipitating events were viral infection (15%), pregnancy (5%) and trauma (5%). All of these patients had persistent symptoms of orthostatic intolerance for more than 6 months duration from the onset of precipitating event.

### Comorbidity

Migraine was the most common co morbidity seen in patients of POTS with JHS (73%) in comparison to 28% in patients of POTS without JHS P< 0.05. All of these patients had a screening transthoracic echocardiogram performed. None of the patients with migraine had any evidence of persistent foramen ovale (PFO) as assessed by a transthoracic echocardiography. No further evaluations to determine the presence or absence of a PFO (such as transesophageal echocardiography) or agitated saline tests were performed.

### Symptoms of POTS

Fatigue, orthostatic palpitations, dizziness and presyncope were similar in patients with and without JHS. However, syncope was significantly higher in patients of POTS with JHS (62%) in comparison to POTS patients without JHS (30%) (P< 0.05).

## Discussion

 Postural orthostatic tachycardia syndrome (POTS) is defined as the presence of symptoms of orthostatic intolerance associated with a heart rate increase of 30 bpm (or rate that exceeds 120 bpm) that occurs within the first 10 minutes of standing or upright tilt, not associated with other chronic debilitating conditions such as prolonged bed rest or the use of medications known to diminish vascular or autonomic tone [12]. The early descriptions of the disorder focused on a group of patients who had been previously healthy until a sudden febrile illness (presumably viral) brought on an abrupt onset of symptoms. Later investigations revealed that POTS is a physiological state most commonly due to inability of the peripheral vasculature to maintain adequate resistance in the face of orthostatic stress, allowing for excessive pooling of blood in the more dependent areas of the body [8,9,13]. The resultant functional decline in circulatory volume elicited a compensatory increase in heart rate and myocardial contractility. While compensatory in mild cases, this mechanism is unable to fully compensate in more severe cases, resulting in a reduction in effective circulation and varying degrees of cerebral hypoperfusion. Later investigations revealed that POTS is not a single condition, but rather a heterogeneous group of disorders resulting in similar physiological state. In 1999 Rowe and colleagues [8] first reported a potential link between what is now referred to the Joint Hypermobility syndrome and POTS. Joint Hypermobility Syndrome is an inherited connective tissue disorder characterized by joint hypermobility, connective tissue fragility, soft 'velvety' skin and variable amounts of tissue hyperextensibility. The condition is assosiated with high incidence of premature varicose veins, easy bruising, diffuse muscle and joint pain as well as pronounced orthostatic acrocyanosis [14]. Subsequently in 2002 Barron et al [7] reported a similar association in a large group of young people suffering from POTS and severe fatigue. In 2003 Gazit et al [9] published a study wherein a group of 48 patients with hypermobility syndrome were compared to a group of 30 healthy control subjects. Each patient underwent a comprehensive series of autonomic tests including tilt table testing, catecholamine level measurement and adrenoreceptor responsiveness. In the hypermobility group 78% tested abnormal as opposed to 10% of control subjects, suggesting that the presence of the condition predisposes the individual to autonomic dysregulation. The authors suggested that the association could have several possible explanations, such as peripheral neuropathy, blood pooling in the lower limbs or impaired central sympathetic control. Other studies have reported that various types of peripheral neuropathies occur more frequently in the joint hypermobility syndrome [15]. It is currently thought that the connective tissue laxity seen in hypermobility patients allows for a greater than normal degree of vascular distensibility leading to an exaggerated amount of blood pooling in the lower extremity during upright posture. As was alluded to previously this then leads to a compensatory tachycardia and increase in cardiac inotropy [9,16]. This could potentially contribute to higher incidence of syncope reported in patients of POTS with preexisting JHS. POTS with preexisting JHS seem to develop symptoms more than a decade earlier than those POTS patients without JHS. While the reasons for this earlier onset are unclear, it is possible that the aforementioned inherent vulnerability of the JHS patients make them more susceptible to environmental stressors than their non JHS peers [7,9]. Migraines were most common comorbidity observed in POTS patients with JHS. This has been seen in other studies as well [17]. Again, the reasons for this are unclear but may potentially relate to abnormal vascular reactivity within the cerebral vasculature. No patient in our study was found to have any evidence of a PFO on 2-D echocardiographic and Doppler evaluation. Syncope was reported in significantly greater number of patients with JHS (62%) as opposed to patients without JHS (30% p< 0.05). Some patients with POTS experience syncope in the absence of significant decline in blood pressure. Sudden increase in cerebrovascular resistance resulting in decline in cerebral oxygenation that occurs in the presence of orthostatic stress has been reported in these patients [18-21].

## Limitations

This was a retrospective analysis of a relatively small patient population. The group of patients reported here included those referred from multiple other centers for a second opinion regarding diagnosis and management. Thus these patients were not true consecutive patients and hence a true incidence of POTS in JHS could not be estimated from this study population. None of the patients with migraine had any evidence of persistent foramen ovale (PFO) as assessed by a transthoracic echocardiography. No further evaluations to determine the presence or absence of a PFO (such as transesophageal echocardiography) or agitated saline tests were performed.

## Conclusion

Patients with POTS and JHS appear to become symptomatic at an earlier age compared to POTS patients without JHS. In addition patients with JHS had a greater incidence of migraine and syncope than their non JHS counterparts.

## Figures and Tables

**Table 1:**
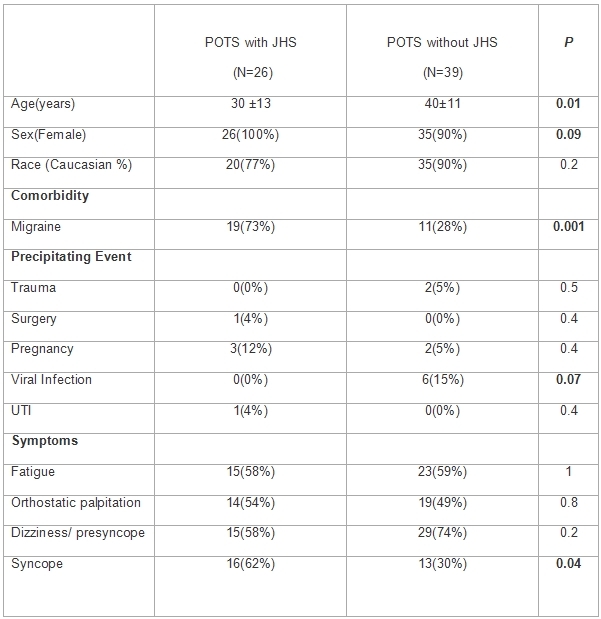
Baseline Clinical characteristics of patients of Postural Tachycardia Syndrome with and without Joint Hypermobility Syndrome
